# Viral Control of Glioblastoma

**DOI:** 10.3390/v13071264

**Published:** 2021-06-29

**Authors:** Nicole Mihelson, Dorian B. McGavern

**Affiliations:** Viral Immunology & Intravital Imaging Section, National Institute of Neurological Disorders and Stroke, National Institutes of Health, Building 10, Room 5N240C, Bethesda, MD 20892, USA; nicole.mihelson@nih.gov

**Keywords:** glioblastoma multiforme, immune evasion, virotherapies, immunotherapeutic strategies

## Abstract

Glioblastoma multiforme (GBM) is a universally lethal cancer of the central nervous system. Patients with GBM have a median survival of 14 months and a 5-year survival of less than 5%, a grim statistic that has remained unchanged over the last 50 years. GBM is intransigent for a variety of reasons. The immune system has a difficult time mounting a response against glioblastomas because they reside in the brain (an immunologically dampened compartment) and generate few neoantigens relative to other cancers. Glioblastomas inhabit the brain like sand in the grass and display a high degree of intra- and inter-tumoral heterogeneity, impeding efforts to therapeutically target a single pathway. Of all potential therapeutic strategies to date, virotherapy offers the greatest chance of counteracting each of the obstacles mounted by GBM. Virotherapy can xenogenize a tumor that is deft at behaving like “self”, triggering adaptive immune recognition in an otherwise immunologically quiet compartment. Viruses can also directly lyse tumor cells, creating damage and further stimulating secondary immune reactions that are detrimental to tumor growth. In this review, we summarize the basic immune mechanisms underpinning GBM immune evasion and the recent successes achieved using virotherapies.

## 1. Introduction

Glioblastoma multiforme (GBM) is intractable and among the most lethal human cancers. GBM is the most common tumor of the adult central nervous system (CNS) and is thought to arise from either neuroglial progenitors or de-differentiated glial cells [1,2,3]. GBM has a median survival of 14 months, and its 5-year survival rate has remained unchanged for nearly half a century, even in the face of new standards of care [4,5,6]. Many theories have been advanced for why GBM is universally lethal and intransigent. First, it harbors a bland mutagenomic profile relative to other cancers, due in part to a few canonical mutations driving oncogenesis as opposed to carcinogens [7]. Second, GBM exhibits vast intra- and inter-individual heterogeneity at both the level of the tumor and immune cells (phenotypically, spatially, temporally, etc.) [8,9] Third, GBM is an intrinsically infiltrative and migratory tumor akin to “sand in the grass,” sitting behind both a blood–brain barrier and inside of a precious-real-estate organ that is unforgiving when it comes to supporting inflammation and immunity.

With the exception of tumor-treating fields (application of low-intensity alternating electric fields) that have increased median survival from 16 to 21 months as well increased 5-year survival from 5% to 13% in exploratory studies [10], virtually none of the many new therapies under investigation have materially prolonged GBM survival [11]. Viral therapy, if further optimized and developed, may be one exception [12]. The idea of using viruses (and other pathogens) to treat GBM was borne from the anecdotes of GBM patients contracting infections in the surgical site and surviving for years. In fact, a 2011 study of 200 GBM patients in Rome found that the 10 who contracted wound infections lived twice as long as their sterile counterparts [13]. One neurosurgeon went on record with *The New Yorker* to say, “If I ever get a GBM, put your finger in your keister and put it in the wound” [14]. Admittedly, this sounds less elegant than checkpoint blockade, but for reasons that will be advanced in this review, such an approach (or an adjacent—more regulated—approach in the vein of deliberate viral infection) is likely to bear more fruit than typical immunotherapeutic strategies. 

## 2. Endogenous Viruses Found in GBM

GBM’s vast intratumoral heterogeneity as well as its rank among the most lethal of all cancers has long prompted queries into its etiology. Viruses have captured the interests of investigators due to their role in the oncogenesis of other cancers, as well as the presence of viral nucleic acid and protein in GBM and the general state of immunosuppression in patients. 

### 2.1. Cytomegalovirus

Human cytomegalovirus (CMV) is a beta herpes virus with a tropism for glial cells. CMV infects 50–90% of the adult population and seroprevalence increases with age [15]. In healthy individuals, CMV is a clinically irrelevant pathogen. In 2002, one of the first CMV studies in GBM showed that 27 of the 27 samples queried by immunohistochemistry and in situ hybridization expressed CMV genomic and protein material [16]. Since then, a plethora of studies have both confirmed and denied these original findings in tumor tissue and blood [17,18,19,20]. Much of the variability has been attributed to assay sensitivity and type, as well as the age of the samples queried [21]. Some groups have also shown that CMV expression was higher in GBM compared to low grade gliomas and epilepsy controls [17], and in newer samples compared with older ones [22]. In studies showing CMV gene products in tumor tissue, their absence in adjacent non-neoplastic tissue begged the question of why lytic phase CMV did not spread. Further, investigators wondered whether CMV’s presence was a product of the chaos in the tumor bed rather than a cause. Indeed, CMV primarily infects quiescent cells, pushes them to G_1_ to induce viral gene expression, and arrests the cell cycle prior to host DNA synthesis in S phase—behavior not entirely conducive with oncogenesis [23]. Furthermore, the prevalence of CMV in nearly 9 out of 10 adults does not match with the prevalence of GBM (1 out of 30,000). Thus, CMV has largely been ruled out as an oncogenic virus, though controversy remains as to whether it is an oncomodulator. According to this theory, CMV does not directly lead to malignant transformation but can perturb cellular processes in ways that promote oncogenic signaling and tumor growth. Treatment of GBM patients with the anti-viral drug Valganciclovir, in addition to standard of care GBM therapy, has been shown to improve median overall survival in newly diagnosed patients [24]. Reverse translational studies in syngeneic murine models revealed that tumor-bearing mice that had been perinatally infected with CMV fared worse than their uninfected counterparts, due in part to increased pericyte recruitment and enhanced angiogenesis in the tumor bed. Treating infected mice with antiviral therapy improved their survival by decreasing PDGF expression and impairing angiogenesis [25]. Collectively, these data support the notion that CMV might promote GBM growth in humans, but additional studies are required to prove this definitively. 

### 2.2. Epstein–Barr Virus

Another oncomodulatory virus with questionable links to GBM is Epstein–Barr Virus (EBV). EBV is a ubiquitous gamma herpes virus that is present in approximately 90% of the adult population and manifests clinically as infectious mononucleosis during its acute lytic cycle. Following acute infection, EBV, like CMV, establishes life-long persistence. In its latency, EBV exists in one of three programs, each hallmarked by the expression of distinct RNA and proteins [26]. One such protein produced during latency programs II and III is LMP1, an oncoprotein that is critical for the carcinogenesis of various subsets of lymphomas and carcinomas [27]. This is in line with data that show EBV’s main cellular reservoir is memory B cells [28,29]. The CNS is also gaining interest as a potential reservoir for EBV latency. Indeed, compliment receptor 2 (CR2), the primary receptor for EBV, is expressed on astrocytes, and upon viral entry, astrocytes demonstrate increased proliferation [30,31]. EBV’s association with a plethora of CNS indications including, but not limited to, ataxia, encephalitis, and demyelinating diseases, as well as its frequent presence in CNS lymphomas, has prompted inquiry into its role in gliomagenesis [32,33]. However, results thus far have been discordant and point to no clear role for EBV in GBM [34].

### 2.3. Human Endogenous Retroviruses

Studies linking human endogenous retroviruses (HERVs) to GBM are sparse, but there appears to be active interest in determining if HERVs contribute to human cancers. HERVs, which are remnants of retroviral germline infections, make up ~8% of the human genome [35]. The majority of HERVs are inactive and unable to replicate. However, certain HERVs, such as those in the HERV-K family, have retained open reading frames, and their viral products are translated during embryogenesis but typically silenced in healthy differentiated cells [36,37]. Due to genetic and epigenetic dysregulation in malignantly transformed cells, high levels of HERV products have been found in the following cancers: breast, lung, prostate, hepatocellular carcinoma, germ cell, melanoma, leukemia, lymphoma, and ovarian, among others [38,39,40,41,42,43,44]. Very recently, one study found that HERV transcripts (mostly from the ERV1 superfamily) were upregulated in GBM compared to adjacent tissue, though notably HERV-K was downregulated [45]. One other study reported that HERV-K could not be detected in GBM [46]. Given these conflicting reports and dearth of information, the role of HERVs in human GBM (if any) remains to be elucidated. 

### 2.4. Exploiting Endogenous Viruses in GBM for Treatment 

One reason why GBM is such an intractable tumor is because it can grow relatively unseen by the adaptive immune system until too late (i.e., when the tumor mass is large) [47]. This relative “invisibility” occurs at various steps of what should otherwise be the typical cancer immunity cycle: (1) release of tumor antigens, (2) antigen presentation by dendritic cells (DCs) and other professional antigen presenting cells (APCs), (3) priming and activation of T cells in secondary lymphoid tissues, (4) infiltration and engagement of the tumor by tumor-specific T cells [48,49]. Given that GBM is thought to be driven primarily by canonical mutations rather than carcinogens or other environmental perturbations, it harbors a far smaller neoantigen burden than other cancers [50,51,52]. Functionally, this means there are less altered self-antigens available for recognition by the immune system in steps 1 and 2 above. Furthermore, the brain lacks a population of migratory APCs to traffic tumor neoantigen to draining lymph nodes. There is also no evidence to date that lymphatic vessels directly contact and drain the brain parenchyma. Instead, parenchymal antigen is thought to drain along the 150 nm wide basement membranes of capillaries and arteries, as well as along exiting neural sheaths [53,54,55]. The little parenchymal antigen that does escape these tight barrier systems would enter the dura mater and potentially drain via its dedicated lymphatics [56,57]. However, we have observed in a murine model of GBM that the drainage of tumor antigen into the cervical lymph nodes is negligible, which impinges on step 3 in the cancer immunity cycle, unless drainage can be enhanced [47]. Lastly, the blood–brain and CSF–brain barriers are designed to limit leukocyte migration into the brain to prevent immunopathology [58,59]. In fact, CNS endothelium only supports the migration of activated leukocytes, and even then, those that extravasate into the perivascular space are sequestered there unless they recognize cognate antigen on perivascular APCs [60,61,62]. The latter would only occur if appropriate tumor antigen drained in the first place. These features impede step 4 of the cancer immunity cycle. In concert, the unique anatomy and immunology of the CNS afford glioblastomas with the ability to remain well ensconced and hidden in the brain. Therefore, it might be incredibly useful to leverage endogenous viral infections against GBM. 

**Figure 1:**
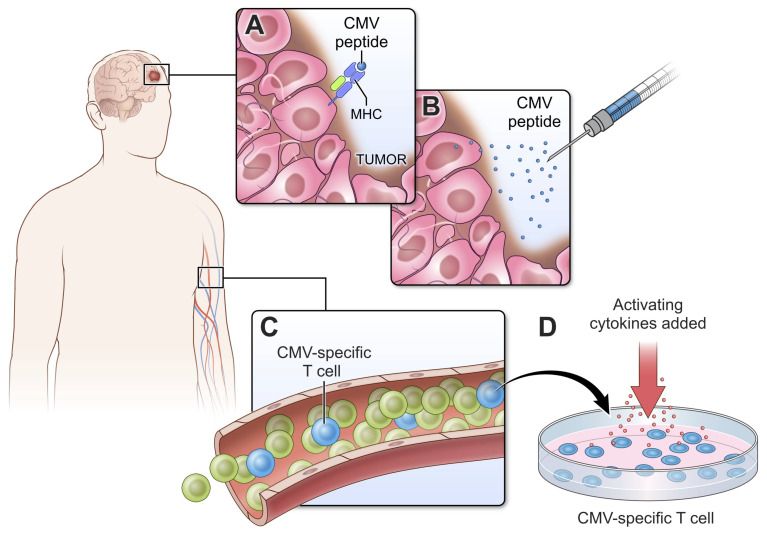
CMV xenogenization of glioblastoma. (**A**) GBM presents CMV peptide on the surface of MHC molecules. (**B**) CMV peptide injected either systemically or intratumorally can be used to activate CMV-specific T cells and route them to the tumor. (**C**,**D**) CMV-specific T cells can comprise up to 10% of the human T cell repertoire and represent a significant contingent of immune cells that can be harnessed for anti-GBM therapy. Many strategies currently involve transferring autologous T cells that have been stimulated ex vivo. Major histocompatibility complex (MHC), cytomegalovirus (CMV).

CMV antigens in infected tumor cells could potentially be exploited as neoantigens worth targeting (Figure 1A). Indeed, given the well-documented subclinical CMV viremia observed in GBM patients, there have been several adoptive T cell transfer and vaccine strategies employing CMV antigens as proxies for GBM-specific antigens and CMV-specific in place of tumor-specific T cells. A notable hallmark of CMV infection is its maintenance of outsized pools of oligoclonal memory T cells, which accumulate over time in a process called “memory inflation”. Impressively, up to one quarter of the total CD8 T cell population of elderly CMV seropositive individuals is CMV-specific [63,64], and in contrast to other latent infections, CMV-specific T cells remain highly functional [65]. Such frequencies of oligoclonal T cells are rarely achieved even with vaccination. This, coupled with the high prevalence of CMV seropositivity in the adult population, makes co-opting this pre-existing pool of “tumor-specific” T cells attractive and pragmatic as a therapeutic. 

The entire approach of rendering GBM antigenically foreign to an otherwise unperturbed host through endogenous viruses predicates on intact viral antigen presentation on the tumor cell surface (Figure 1A). Though CMV has been detected in GBM, these assays do not test nor guarantee that CMV peptides are presented by major histocompatibility complexes (MHC), and there is adequate reason to doubt the extent to which appropriate antigen presentation occurs. Over half of human GBM specimens show loss of HLA class I antigens, and of the remaining fraction, half show faint staining, leaving only 20% of tumors with robust, intact membrane-bound MHC I expression [66,67]. The assessment of MHC I expression should thus be routine in GBM care and should occur prior to the start of CD8 T cell-based immunotherapies. The absence of MHC can be irreversible, as in the case of loss of heterozygosity (LOH) mutations at 6p21 and 15q21, loci corresponding to HLA class I and β2-microglobulin (β2m)*,* respectively, which are critical components of the MHC molecule [68]. This occurs in almost half of GBM cases, and therapeutic strategies for these patients should be independent of MHC class I antigen presentation and CD8+ T cell engagement [68]. In other cases, the absence of surface MHC class I can be reversed with appropriate cytokine stimulation (e.g., type I and II interferons) provided the HLA-A, HLA-B, and HLA-C gene loci are intact [69]. 

Patients with intact MHC loci might benefit from efforts underway to activate CMV-specific T cells and promote their subsequent engagement of brain tumors by injecting CMV peptides, and early reports demonstrate the safety and feasibility of this approach (Figure 1B) [70,71]. Other methods to activate CMV-specific T cells (Figure 1C) have included the transfer of autologous DCs pulsed ex vivo with CMV lower matrix protein pp65 as an adjuvant to standard of care chemoradiation. Across three sequential, independently conducted clinical trials, nearly one third of GBM patients receiving pp65 DCs became “exceptional long-term survivors” [72,73]. More recent efforts to treat patients with autologous CMV-specific T cells activated and expanded ex vivo (Figure 1D) demonstrated safety as an adjuvant therapy for primary and recurrent GBM and the potential to extend survival in part via epitope spreading [74,75,76]. The latter is particularly important given how “quiet” the adaptive immune response would otherwise be. GBM also has a well-documented history of antigenic escape following therapeutic pressure [77]. Therefore, harnessing anti-viral immunity to wage an effective GBM response will likely require polyclonal T cell responses as well as epitope spreading to other antigens. The functional attributes of CMV-specific CD8 T cells are also important. To improve the polyfunctionality of therapeutic T cells, investigators recently combined the transfer of autologous CMV pp65-specific CD8+ T cells with pp65 RNA-loaded DCs. This combination improved frequencies of IFNγ, TNFα and CCL3 positive, polyfunctional CMV-specific T cells, which correlated with an increased overall survival [78]. However, it remains to be determined how these functional attributes influenced the subsequent antitumor response. 

The strategies summarized in Figure 1 aim to combat the low immunogenicity of GBM and mount an adaptive antitumor immune response by harnessing CMV xenogenization of the tumor [79]. The efficacy of these strategies depends on there being sufficient cognate peptide-MHC I complexes on the surface of tumor cells for subsequent CD8+ T cell engagement and killing. However, MHC I loss is irreversible in nearly half of GBM patients [68], and in the majority of the remaining half, MHC is significantly downregulated [66,67,69]. In patients with a loss of MHC I expression, immunotherapeutic strategies must rely on leukocyte–tumor interactions that are independent of MHC (e.g., NK cells or antibodies). On the other hand, patients with inducible MHC I expression should be treated with dual approaches that first upregulate tumor antigen presentation (e.g., with type I or II interferons) and then promote cytolytic engagement of the tumor by anti-tumor (viral) CD8+ T cells. 

## 3. Oncolytic Viral Therapy

**Figure 2:**
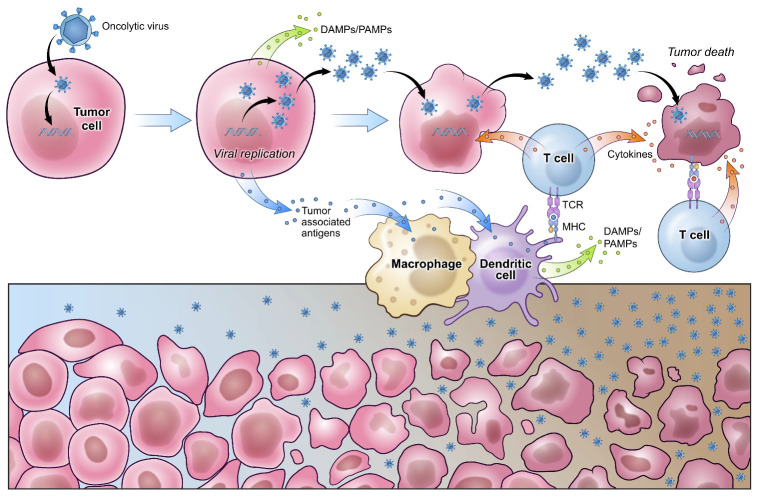
Viral oncolysis of glioblastoma. Oncolytic viruses are either naturally selected or engineered to preferentially infect tumor cells. Upon entry, viruses co-opt tumor cell machinery to support their own replication and lyse tumor cells in the process. Viral progeny are then released and continue infecting surrounding tumor cells. Secondary immune reactions develop in response to damage- and pathogen-associated molecular patterns (DAMPs and PAMPs), breaking immune tolerance against GBM. Increased antigen presentation facilitates the generation of both anti-viral and anti-tumor T cells that help destroy tumor cells. T cell receptor (TCR), major histocompatibility complex (MHC).

Oncolytic viruses (OVs) are naturally occurring or genetically engineered viruses that selectively infect and lyse tumor cells, disseminating to adjacent malignant cells to repeat the process. OVs exploit the inherent dysregulation within the cancer cell—from cell signaling abnormalities to incapacitated antiviral machinery—as well as the cancer’s own immune evasion pathways to preferentially infect and reproduce in neoplastic cells [80]. For this reason, oncolytic viruses do not necessarily rely on neoantigens at the tumor cell surface for their selective infection of tumor cells. OVs orchestrate antitumor activity through two distinct but related mechanisms. The first is their direct lysis of tumor cells, and the second is the concomitant innate and adaptive response mounted in response to damaged-associated molecular patterns (DAMPs) and viral pathogen-associated molecular patterns (PAMPs) released from dying tumor cells (Figure 2). The latter is thought to break immune tolerance against GBM. In fact, the molecular signals and cytokines induced by infection with OVs activate and attract a variety of APCs, as well as innate and adaptive immune cell subsets. Remarkably, the induction of a pleiotropic anti-GBM response by OVs has promoted survival in a subset of GBM patients. 

Among the most promising candidates is a prototype oncolytic poliovirus (PV) recombinant, PVSRIPO—a modified poliovirus lacking neurovirulence [81]. The tropism of PVSRIPO is based on CD155, which is a type I transmembrane glycoprotein broadly upregulated on a variety of tumor cells, but not on normal adult neural cells, and on APCs [82]. The infection of DCs by PVSRIPO does not lyse them. Rather, it induces a strong type I interferon response, enhances antigen presentation, and promotes the ability of DCs to prime tumor-specific T cells that traffic to the tumor microenvironment [83,84]. In a recent Phase 2 dosing study, PVSRIPO delivered intratumorally via catheter modestly increased survival relative to a historical control group from 11.3 to 12.5 months. However, more impressive was the fact that survival plateaued at 21% in PVSRIP patients at 24 months and 36 months compared to 14% and 4%, respectively, in historical controls [12]. These data suggest that a robust anti-tumor immune response was sustained in patients who responded to treatment, although additional studies into the HLA status and anti-tumor immune response in surviving patients are warranted. It will also be important to determine whether PVSRIPO promoted the release of hidden tumor neoantigens. Though most patients experienced adverse events related to brain inflammation, a concern with OVs, edema was successfully managed in all cases. Some potential confounding variables in this exciting trial include the younger age of study participants as well their higher initial gross total resection rates and lower rates of prior bevacizumab (anti-VEGF) therapy than historical controls. 

In other OV trials, subsets of patients have similarly experienced durable clinical responses. Following intratumoral inoculation of a tumor-specific adenovirus, DNX-2041, 20% of patients were alive at 36 months [85]. Radiographic data in responders suggested immunogenic cell death, which is hypothesized to be the mechanism of action with OVs. G47∆, a third-generation type I Herpes Simplex Virus (HSV-1) developed in Japan, received “Sakigake” breakthrough status and is expected to receive fast-track drug approval soon. Recent preliminary data from a Phase II study following six intratumoral injections of G47∆ showed a one year survival rate of 92.3% relative to the historical control rate of 15% [86]. HSV-1 possesses a few advantageous properties. First, it is highly proliferative and thus can achieve high titers. Second, it does not integrate into the host genome. Third, anti-HSV-1 agents to which G47∆ is hypersensitive are readily available, providing a safety break if needed [87]. Despite the capacity of HSV-1 to infect a variety of cells, the deletion of ICP6, a ribonucleotide reductase (RR) required for viral DNA synthesis, in G47∆ renders the virus dependent on abundant RR in the cells it infects. Tumor cells, but not normal cells, express sufficient compensatory levels of RR to support G47∆ replication [88]. 

Another virus that has attracted attention for its neurotropism is Zika Virus (ZIKV). It was recently reported that ZIKV preferentially lyses patient-derived glioma stem cells (GSCs) over differentiated GBM cells or human neural precursors, due in part to increased surface integrin alpha chain V expression [89,90]. GSCs are thought to drive tumor growth, immune evasion, and recurrence given their self-renewing multipotent nature, selective therapeutic resistance, and capacity to co-opt angiogenesis to self-sustain [1]. The use of ZIKV in GBM is still in the preclinical phase.

**Figure 3:**
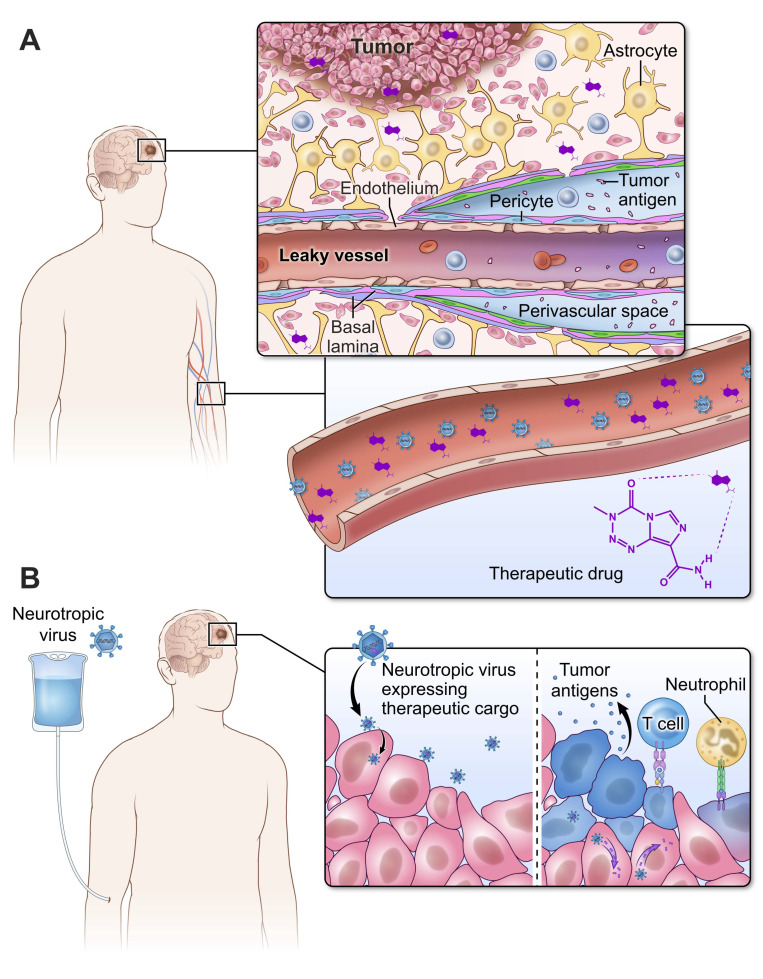
The blood–brain barrier (BBB) makes it challenging to deliver drugs to GBM. (**A**) Approximately 20% of the delivered concentration of anticancer therapeutics reaches the tumor bed despite the presence of leaky vessels. The BBB limits the degree to which therapeutics can access GBM. (**B**) Neurotropic viruses are naturally selected biologic agents that can traverse the BBB and infect the tumor bed. These viruses can be engineered to carry therapeutic cargo that is delivered directly to tumor cells, stimulating innate and adaptive immune responses.

OVs offer the promise of not only warming an otherwise “cold” tumor niche, but also of delivering therapeutic cargo encoded in the virus. This is of particular importance in GBM since the various barrier systems of the CNS passively and actively (via efflux pumps) work to prevent the infiltration of delivered drugs. Indeed, drug concentrations in the tumor microenvironment can be less than 20% of their concentration in the plasma (Figure 3A) [91]. Arming OVs with immunostimulants provides another opportunity for this new class of therapeutics to break immune tolerance against GBM and control cargo delivery both spatially and temporally (Figure 3B). In pre-clinical studies using a fatal genetic model of GBM, an oncolytic HSV encoding IL-12 resulted in an unprecedented degree of protection and a 30% cure [92]. 

## 4. Concluding Remarks

GBM remains one of the deadliest cancers, and its long-term survival rate has not changed in the last 50 years. There is a plethora of reasons for GBM’s recalcitrance and lethality, a few of which include its striking heterogeneity, subset of stem cells, and capacity to induce systemic immune suppression. There are also significant limitations in the capacity of the brain to detect GBM antigens and support afferent as well as efferent immune responses. Few interventions can overcome all these hurdles except for virotherapy. Further studies are warranted to better understand the mechanisms by which these virotherapies work and improve upon their efficacy in all GBM patients. It will be important to map out determinants of long-term durable anti-tumor immune responses and identify other immunomodulatory therapies that provide synergistic combinatorial effects. Nonetheless, the rapid development of viral therapies for GBM starting at the turn of this century may ultimately have the greatest impact on improving life expectancy for GBM patients.

## Data Availability

Not applicable.
